# Latin American ophthalmology practitioner’s perception on current COVID-19 pandemic

**DOI:** 10.5935/0004-2749.202100103

**Published:** 2021

**Authors:** Nora Lucía Oliva Castillo, Evelyn del Busto Wilhelm, Martin Arturo Zimmermann Paiz, Ana Marissa Ordóñez Rivas, Nancy Carolina Quezada del Cid, Verónica Yaneth Burgos Elías, Edison Leonardo Montes Villalva

**Affiliations:** 1 Benemérito Comité Pro-Ciegos y Sordos de Guatemala. Hospital de Ojos y Oídos “Dr. Rodolfo Robles Valverde”. Instituto de Ciencias de la Visión. Unidad de Oftalmología Pediátrica, Estrabismo y Neuro-Oftalmología “Dra. Ana María Illescas Putzeys.”

**Keywords:** Pandemics, Ophthalmology, Health care survey, Latin America, SARS-Cov2, Coronavirus infections, COVID-19, Pandemias, Oftalmologia, Pesquisas sobre serviços de saúde, América Latina, SARS-CoV2, Infecções por coronavirus, COVID-19

## Abstract

**Purpose:**

To assess the perception of Latin American ophthalmology practitioners regarding coronavirus disease 2019 (COVID-19) exposure risk, knowledge about personal protection measures, and prioritization of patients.

**Methods:**

Self-administered voluntary anonymous survey (Google Drive forms) was distributed via text message to ophthalmology practitioners from May 01 to May 05, 2020.

**Results:**

Three hundred seventy-one practitioners (45% response rate) comprising 118 (27.6%) residents, 111 (40.5%) ophthalmologists, and 142 (32.8%) sub-specialists completed the survey. Among them, 106 (32.6%) thought that they were at a high risk of acquiring COVID-19 during the course of work. Furthermore, 273 (69.1%) believed that the current guidelines were insufficient to identify COVID-19 patients. The survey also revealed that 265 (59.5%) were not trained to use personal protective equipment (PPE), and even with its correct use, 341 (91.5%) still felt that they were at risk of acquiring COVID-19. Moreover, 80% of the respondents were of the view that staff members were not knowledgeable about national protocols for attending COVID-19 patients. However, only 9 (2%) considered changing their profession to ameliorate COVID-19 contagion risk.

**Conclusion:**

This survey has revealed the issues faced by ophthalmology practitioners in Latin America during their routine practice. These concerns and anxiety about COVID- 19 pandemic seem to be the same worldwide. It is important to reinforce the confidence of ophthalmology practitioners on current guidelines for attending COVID-19 patients. It is also necessary to conduct training programs on PPE usage and ensure that PPE items are available at all times to enhance the quality of care and minimize the spread of the disease.

## Abstract (pt)

**Objetivos:**

Avaliar a percepção do risco de exposição da Doença de Coronavírus 2019 (COVID-19), conhecimento sobre medidas de proteção pessoal entre os profissionais de oftalmologia latino-americanos e priorização de pacientes com Covid-19.

**Métodos:**

Pesquisa anônima voluntária autoadministrada (formulários do Google Drive) distribuída por mensagem de texto para profissionais de oftalmologia em 1º a 5 de maio de 2020.

**Resultados:**

Trezentos e setenta e um profissionais completaram a pesquisa (taxa de resposta de 45%), composta por 118 residentes (27,6%), 111 oftalmologistas (40,5%) e 142 subespecialistas (32,8%). 106 profissionais (32,6%) sentiram-se em alto risco de adquirir o COVID-19 no trabalho. 273 (69,1%) acreditavam que as diretrizes atuais não são suficientes para identificar os pacientes com COVID-19. 265 (59,5%) não tinham treinamento para usar os equipamentos de proteção individual (EPI) e, mesmo com seu uso correto, 341 (91,5%) ainda se sentiram em risco de adquirir COVID-19. 80% consideraram que a equipe de trabalho não tem conhecimento de protocolos nacionais para o atendimento aos pacientes com COVID-19. Apenas 9 dos profissionais (2%) consideraram mudar a profissão para minimizar o risco de contágio por COVID-19.

**Conclusão:**

Esta pesquisa mostra a escassez de pessoal e treinamento específico que os praticantes de oftalmologia na América Latina enfrentam em sua prática diária. Essas preocupações e ansiedade parecem ser as mesmas em todo o mundo com a pandemia de COVID- 19. É importante reforçar a confiança dos profissionais de oftalmologia nas diretrizes atuais de atendimento ao paciente com COVID-19 e também disponibilizar programas de treinamento sobre o uso de EPI e também itens de EPI disponíveis em todos os momentos para garantir a qualidade do atendimento e a disseminação mínima da doença.

## INTRODUCTION

An outbreak of pneumonia of unknown etiology was reported by the Health Commission of Hubei, China, on December 31, 2019^(1)^. In January 2020, severe acute respiratory syndrome coronavirus 2 (SARS-CoV-2) was identified as the causative agent of the severe pneumonia, which is now known to be a complication of the coronavirus disease 2019 (COVID-19)^(2)^. Since then, COVID-19 has continued to spread, and the World Health Organization (WHO) declared it as a pandemic on March 11, 2020^(3)^. The disease is highly infectious, and spreads via respiratory droplets and possibly from contaminated surfaces^(4)^.

Ophthalmology practitioners are susceptible to the infection because of their proximity with patients during the examination^(5)^. This study aims to assess the perception of COVID-19 exposure risk, knowledge about personal protection measures, and the prioritization of patients in ophthalmological practice in some Latin American countries.

## METHODS

We conducted a self-administered, voluntary, anonymous survey using a questionnaire that comprised 17 questions (Google Drive forms) (Table 1). The forms were distributed via text messages to 777 ophthalmology practitioners working in México, Guatemala, El Salvador, Honduras, Colombia, Ecuador, Perú, and Argentina. The survey was open from May 01 to May 05, 2020. The data were extracted from Google Forms, and a database was created using the Statistical Package for the Social Sciences (IBM^®^SPSS^®^ Statistics 2019 Chicago, IL USA). Student’s t-test was employed to determine the differences among the responses from different countries. A p value <0.05 was considered statistically significant; measures of descriptive statistics were also taken.

**Table 1:** Questions and answers included in the survey

Question	Answer options
1. Which ophthalmology position do you perform?	Resident Ophthalmologist Sub-specialist
2. How much exposure to COVID-19 do you consider having during your ophthalmology practice?	1 through 5 (1 =no risk, 5= high risk)
3. Do you consider that current research and guidelines are enough to allow you to identify COVID-19 patients during your	Yes
ophthalmology practice?	No
4. Which patients conditions do you consider a priority for attention during this pandemic time?	Conditions listed in table 3
5. Did you receive suitable training to use PPE?	Yes No
6. Do you consider that the whole medical and administrative staff at your workplace has knowledge of national protocols	Yes
to COVID-19 patients attention?	No
7. Do you consider that the whole medical and administrative staff at your workplace has knowledge of institutional protocols	Yes
to COVID-19 patients attention?	No
8. Do you have available PPE?	Yes No
9. PPE is provided at your workplace?	Yes No Partially
10. Even with PPE usage, do you consider yourself at COVID-19 infection risk?	Yes No
11. Which of the following PPE items do you currently wear in your ophthalmology practice?	Items listed in table 2
12. Do you consider telemedicine to be feasible in ophthalmology practice?	Yes No
13. Do you consider periodical COVID-19 screening should exist among health care providers owing to asymptomatic cases?	Yes No
14. Is shift work being implemented at your workplace to ensure social distancing?	Yes No
15. Do you have knowledge about where to refer COVID-19 cases?	Yes No
16. Do you consider changing your profession to avoid exposure to COVID-19?	Yes No
17. Do you consider yourself at high risk of complications if you acquire COVID-19?	Yes No

Seventeen choice questions distributed by text message that ophthalmology practitioners voluntarily answered.

## RESULTS

Three hundred seventy-one practitioners, including 40 Mexicans, 128 Guatemalans, 26 Salvadorans, 39 Hondurans, 8 Colombians, 90 Ecuadorians, 9 Peruvians, and 31 Argentinians completed the survey (45% response rate). The respondents included 118 (27.6%) residents, 111 (40.5%) ophthalmologists, and 142 (32.8%) sub-specialists. Among them,106 (32.6%) expressed being at a high risk of acquiring COVID-19 during the course of their work (assessed on a 1-5 scale, with 1 being low risk and 5 being high risk). Furthermore, 273 (69.1%) believed that current guidelines were inadequate to identify COVID-19 patients.

Two hundred sixty-five respondents (59.5%) were not trained to use personal protective equipment (PPE). Another 297 (83.1%) indicated that they used PPE, with 34.7% receiving it entirely and 53.8% receiving it partially from their workplace. Even with the correct usage of PPE, 341 (91.5%) felt that they were at risk of acquiring COVID-19. Moreover, 253 (70.1%) were of the opinion that they were not at risk of complications even if infected. The PPE items used are summarized in table 2.

**Table 2:** PPE items used by ophthalmology practitioners

PPE item	México n=40	Guatemala n=128	El Salvador n=26	Honduras n=39	Colombia n=8	Ecuador n=90	Perú n=9	Argentina n=31
Hand washing	40 (100)	128 (100)	26 (100)	39 (100)	8 (100)	88 (97.8)	8 (88.9)	30 (96.8)
Auto filtering mask	34 (85)	119 (93)	24 (92.3)	38 (97.4)	8 (100)	87 (96.7)	8 (88.9)	22 (71)
Slit lamp shield	35 (87.5)	93 (72.7)	21 (80.8)	7 (2.6)	6 (75)	61 (67.8)	5 (55.6)	26 (83.9)
Ocular protection	37 (92.5)	92 (71.9)	20 (76.9)	29 (74.4)	7 (87.5)	80 (88.9)	5 (55.6)	26 (83.9)
Gloves	26 (65)	82 (64.1)	9 (34.9)	29 (74.4)	6 (75)	72 (80)	5 (55.6)	26 (83.9)
Facial protection	23 (57.5)	59 (46)	19 (73.1)	17 (43.6)	5 (62.5)	47 (52.2)	1 (11.1)	17 (54.8)
Disposable gown	9 (22.5)	45 (35.2)	8 (30.8)	15 (38.5)	8 (100)	52 (57.8)	4 (44.4)	25 (80.6)

Numbers in the body of the table indicate the number of participants from each country that wore a specific PPE item.

The ocular conditions which are considered as a priority for attention are summarized in table 3.

**Table 3:** Ocular conditions considered as emergencies by ophthalmology practitioners

Ocular condition	México n=40	Guatemala	El Salvador n=26	Honduras n=39	Colombia n=8	Ecuador n=90	Perú n=9	Argentina	P value
Ocular trauma	39 (97.5)	123(96.1)	26 (100)	37 (94.9)	8 (100)	89 (98.9)	9 (100)	29(93.5)	<0.05
Ulcerative keratitis	37 (92.5)	126 (98.4)	25 (96.2)	38 (97.4)	8 (100)	88 (97.8)	9 (100)	30 (96.8)	<0.05
Acute glaucoma	35 (87.5)	124 (96.9)	25 (96.2)	37 (94.9)	8 (100)	83 (92.2)	9 (100)	31 (100)	<0.05
Endophthalmitis	39 (97.5)	123 (96.1)	25 (96.2)	36 (92.3)	8 (100)	81 (90)	9 (100)	31 (100)	<0.05
Corneal burn	34 (85)	120 (98.8)	22 (84.6)	31 (79.5)	8 (100)	83 (92.2)	8 (88.9)	25 (80.6)	<0.05
Corneal/conjunctival foreign body	31 (77.5)	113 (88.3)	21 (80.8)	37 (94.9)	8 (100)	79 (87.8)	6 (66.7)	21(67.7)	<0.05
Retinal detachment	20 (80)	113 (88.3)	23 (88.5)	33 (84.6)	8 (100)	75 (83.3)	9 (100)	30 (96.8)	<0.05
Painful red eye	31 (77.5)	104 (81.3)	20 (76.9)	30 (76.9)	8 (100)	67 (74.4)	5 (55.6)	29 (93.5)	<0.05
Post-operative period	34 (85)	94 (73.4)	23 (88.5)	27 (69.2)	0 (0)	56 (62.2)	5 (55.6)	18 (58.1)	<0.05
Infection requiring intravenous antibiotic	25 (62.5)	92 (71.9)	13 (50)	29 (74.4)	5 (62.5)	45 (50)	4 (44.4)	25 (80.6)	<0.05
Retinopathy of prematurity	50 (20)	87 (68)	9 (34.6)	23 (59)	8 (100)	56 (62.2)	9 (100)	23 (74.2)	<0.05
Acute visual acuity loss	25 (62.5)	80 (62.5)	9 (34.6)	19 (48.7)	7 (87.5)	37 (41.1)	3 (33.3)	24 (77.4)	<0.05
Leukocoria	23 (57.5)	66 (51.6)	7 (26.9)	17 (43.6)	5 (62.5)	29 (32.2)	3 (33.3)	14 (45.2)	<0.05
Suspected tumoral lesion	17 (42.5)	64 (50)	6 (23.1)	13 (33.3)	5 (62.5)	27 (30)	3 (33.3)	14 (45.2)	<0.05
Diplopia	15 (37.5)	51 (39.8)	8 (30.8)	19 (48.7)	6 (75)	31 (34.4)	1 (11.1)	20 (64.5)	<0.05
Unilateral red eye, no secretion	11 (27.5)	45 (35.2)	9 (34.6)	12 (30.8)	3 (37.5)	23 (25.6)	1 (11.1)	16 (51.6)	<0.05
Acute strabismus/nystagmus	11 (27.5)	50 (39.1)	3 (11.5)	16 (41)	6 (75)	21 (23.3)	4 (44.4)	23 (74.2)	<0.05
Diabetic retinopathy	17.5 (7)	34 (26.6)	4 (15.4)	16 (41)	4 (50)	24 (26.7)	3 (33.3)	7 (22.6)	<0.05
Stye/chalazion/blepharitis	2 (2.5)	15 (11.7)	3 (11.5)	3 (7.7)	0 (0)	11 (12.2)	1 (11.1)	0 (0)	<0.05
Amblyopia	2 (5)	15 (11.7)	1 (3.8)	3 (7.7)	0 (0)	5 (5.6)	2 (22.2)	4 (12.9)	0.1
Cataract surgery	0 (0)	3 (2.3)	0 (0)	4 (10.3)	2 (25)	5 (5.6)	0 (0)	1 (3.2)	1
Refraction, contact lenses prescription, refractive surgery	0 (0)	0 (100)	0 (0)	1 (2.6)	0 (0)	3 (3.3)	0 (0)	0 (0)	0.2

Student’s T-test.

Numbers indicated in the body of the table indicate the number of participants from each country who mentioned a specific condition that urgently required treatment.

The ophthalmology practitioners felt that the medical and administrative staff were not knowledgeable about the national (80%) and institutional (71.7%) protocols for attending COVID-19 patients. Among the respondents, 308 (80.9%) knew where to refer suspected cases.

Shift-based work was used in 303 instances (79.8%) to ensure social distancing. Three hundred twenty respondents (96.7%) thought that routine testing of healthcare providers is necessary to detect asymptomatic cases.

One hundred sixty-seven (50.4%) considered telemedicine to be suitable in ophthalmology practice.

Only 9 (2%) thought of changing their profession to ameliorate COVID-19 contagion risk.

## DISCUSSION

Healthcare professionals are generally at high risk of COVID-19 infection, and for ophthalmology practitioners, the risk is even higher^(6,7)^. In the countries included in this survey, over 25,000 healthcare providers have tested positive and some of them have succumbed to the illness^(8-14)^.

Minocha et al.^(15)^ conducted a similar survey in the United Kingdom and discovered that out of the 100 ophthalmology practitioners surveyed, 80 (80%) felt that they were at high risk of acquiring COVID-19 at work. In our survey, 106 (32.6%) expressed being at high risk of infection. Similar to our findings, the results of Minocha et al.^(15)^ also suggested an absence of training to use PPE; hence, training programs are the need of the hour. In this survey, 265 (59.5%) had no training to use PPE, and 345 respondents (91.5%) felt that they were at risk of acquiring COVID-19 even with PPE usage.

The WHO and The United States’ Centers for Disease Control and Prevention (CDC) recommend the use of full PPE while evaluating patients suspected to have COVID-19, including medical mask, gown, gloves, eye protection, and hand hygiene. For the examination of patients without COVID-19 symptoms, they suggest hand hygiene and PPE according to standard precautions and risk assessment^(16)^. However, several studies have found that the proportion of asymptomatic COVID-19 patients is variable (5%-80%), and hence, it is difficult to decide the circumstances under which full PPE is required^(17)^. In this survey, PPE was completely available at the institution only for 34.7% of the practitioners, partially available for 40.8%, and not available at all for 24.5% of the practitioners.

The fact that no PPE is available for some ophthalmology practitioners is an issue of concern. Statistically significant differences existed among the PPE items used in the different countries that were assessed, which might be because standardized policies are lacking.

Furthermore, with regard to asymptomatic COVID-19 cases, 96.7% of the ophthalmology practitioners who participated in this survey felt that routine testing of healthcare providers is essential to detect the infected individuals and reduce the contagion risk.

In a survey of 62 United States’ vitreoretinal surgery fellows, Khan et al.^(18)^ found that 95.1% used a surgical mask for all patient contacts and 53.2% ensured that the patients wore face masks as a risk mitigation strategy; however, 11.3% had no access to a N95 respirator. In this survey, 90.5% of the ophthalmology practitioners used auto filtering masks for patient contact. Nonetheless, we did not assess the use of face masks on patients or the specific availability of N95 respirators.

As already mentioned, ophthalmologists are at risk of COVID-19 infection. The close proximity between the patients and the doctors during the examination, the presence of tears during anesthesia and dilation, and the potential aerosols from “air puff” tonometry pose a high contagion risk. Conjunctivitis is present in 0.8%-5.2% of the COVID-19 patients, which can be the presenting symptom, and the virus may even be present in tears and conjunctival secretions^(19)^.

Direct contact with the ocular surface and the mucosal membrane during routine ophthalmic investigations may be associated with a high risk of infection when a slit lamp examination is performed. The presence of a physical barrier between the doctors and the patients is advisable to prevent droplet transmission^(19)^. In this survey, an average of 65.7% of the respondents reported the use of this type of protection, and it was the highest in México and the lowest in Honduras (87.5% vs 2.6%, *p*<0.05).

We found statistically significant differences among the ocular conditions that the ophthalmology practitioners considered as emergencies; only amblyopia, cataract surgery, and refraction/contact lens prescription/ refractive surgery were agreed to be non-urgent conditions or procedures. This opinion reflects the following statement of the American Academy of Ophthalmology: “The Academy recognizes that “urgency” is determined by physician judgment and must always take into account individual patient medical and social circumstances”^(20)^.

Nowadays, the CDC recommends telemedicine instead of live clinical evaluations. The patients are also looking for digital care, which is reflected in the fact that some leading telehealth platforms have reported a 257%-700% hike in virtual patient visits^(21)^. In this survey, 50.4% of the Latin American ophthalmology practitioners agreed that telemedicine is a possibility in their practice although many eye pathologies were classified as emergencies that warranted face-to-face evaluation.

It is noteworthy that only 20.04% of the surveyed respondents believed that the medical and administrative staff had knowledge of the national protocols for attending COVID-19 patients. Additionally, only 28.3% were of the opinion that there was a general knowledge of the institutional protocols; hence, it seems prudent to provide more information and training. Taking measures based on evidence-supported guidelines is a priority.

Our survey has revealed the key issues faced by the Latin American ophthalmology practitioners in their routine practice. These concerns and the anxiety related to the pandemic seem to be the same worldwide. It is imperative to reinforce the confidence of the ophthalmology practitioners on the current guidelines for attending COVID-19 patients. Furthermore, training programs on PPE usage should be conducted, and PPE items should be made available at all times to ensure the quality of care and curtail the spread of the disease.
